# The Preoperative Predictive Factors for Pathological T3a Upstaging of Clinical T1 Renal Cell Carcinoma

**DOI:** 10.3390/diagnostics9030076

**Published:** 2019-07-15

**Authors:** Shinji Fukui, Makito Miyake, Kota Iida, Kenta Onishi, Shunta Hori, Yosuke Morizawa, Yoriaki Kagebayashi, Kiyohide Fujimoto

**Affiliations:** 1 Department of Urology, Nara Medical University, 840, Shijo-cho, Kashihara, Nara 634-8521, Japan; 2 Department of Urology, Nara Prefecture General Medical Center, 897-5, Shichijo-nishi machi 2 chome, Nara, Nara 630-8581, Japan

**Keywords:** renal cell carcinoma, upstaging, AST/ALT ratio

## Abstract

We aimed to determine the oncological outcomes of patients with clinical T1 renal cell carcinoma (RCC) upstaged to pathological T3a and to identify the preoperative predictive factors for upstaging. We retrospectively reviewed 272 patients with clinical T1 RCC who underwent surgical treatment. Thirty-three patients (12%) were upstaged to pathological T3a. These patients had a significantly larger tumor size on computed tomography (*p* < 0.0001), a higher aspartate aminotransferase (AST)/alanine aminotransferase (ALT) ratio (*p* = 0.037), and an elevated c-reactive protein (CRP) level (*p* = 0.014) preoperatively compared with those with pathological T1 RCC. On multivariate analysis, tumor diameter was the only significant preoperative predictive factor for upstaging [hazard ratio (HR), 3.61; 95% confidence interval (CI), 1.32–9.84; *p* = 0.01]. The AST/ALT ratio tended to be a preoperative predictive factor for upstaging, although it was not significant (HR, 2.14; 95% CI, 0.97–4.73; *p* = 0.06). Pathological T3a upstaging occurred in 25% of those with a tumor diameter ≥30 mm and a preoperative AST/ALT ratio ≥1.1. There was a significant correlation between pathological T3a upstaging and the number of preoperative risk factors (*p* = 0.0002). The preoperative tumor diameter and serum AST/ALT ratio can be predictive factors for pathological T3a upstaging in patients with clinical T1 RCC.

## 1. Introduction

Clinical staging is essential for treatment decision-making in patients with renal cell carcinoma (RCC). The selection of the surgical technique (partial or radical nephrectomy) often depends on the clinical T stage of the tumor; T1 and T2 tumors are classified according to the tumor size, whereas T3a tumors are defined based on the presence of peripheral fat invasion, renal sinus fat infiltration, or renal vein extension, regardless of the tumor size. However, microscopic perirenal invasion, renal sinus fat infiltration, and renal vein extension can be missed during contrast-enhanced computed tomography (CT) imaging, occasionally leading to the pathological upstaging of a clinical T1 tumor to pathological T3a. Several previous studies have reported a poor prognosis of patients with RCC with cT1 tumors upstaged to pT3a when compared with a prognosis of patients with pathological T1 tumors [1,2,3,4]. This study investigates the oncological outcomes of pathological T3a upstaging and identifies the preoperative predictive factors for upstaging in patients with cT1 RCC.

## 2. Materials and Methods

We retrospectively reviewed the records of all patients with non-metastatic clinical T1 renal tumors who underwent partial nephrectomy (PN) or radical nephrectomy (RN) at Nara Medical University and Nara Prefecture General Medical Center between January 2000 and December 2017. Institutional review board approval was obtained for this study. Two hundred seventy-two patients (196 men and 76 women) diagnosed with RCC who had complete records of preoperative demographics and postoperative outcomes were included for further analysis. Patients who underwent renal replacement therapy, such as hemodialysis or peritoneal dialysis, and those with liver disease during treatment were excluded. We also excluded patients with non-RCC pathology, bilateral or multiple renal tumors, and von Hippel–Lindau disease. The clinical T stage was assessed with contrast-enhanced CT.

We evaluated the following preoperative characteristics: Age at the time of operation, gender, body mass index (BMI), tumor laterality, clinical T stage, RENAL nephrometry score, and tumor diameter upon contrast-enhanced CT, as well as serum aspartate aminotransferase (AST), alanine aminotransferase (ALT), and c-reactive protein(CRP) levels. The AST/ALT ratio was also calculated. Surgical and histopathological data included surgical technique (PN, RN, laparoscopic surgery, or open surgery), tumor histology, Fuhrman grade, and the presence of microscopic venous invasion and/or lymphatic invasion. The surgical technique was determined by discussion among experienced urologists after taking into account patient comorbidities, tumor size, tumor location, and intraoperative findings. Lastly, outcomes were measured using overall survival (OS), cancer-specific survival (CSS), and recurrence-free survival (RFS).

Data were analyzed using JMP^®^ 13 software (SAS Institute Inc., Cary, NC, USA). The Mann–Whitney, chi-square, and Fisher’s exact tests were used to compare the pT3a upstage and pT1 groups. OS, CSS and RFS was estimated using Kaplan-Meier method. Cox proportional hazards regression was used to examine variables associated with pT3a upstaging. A *p*-value < 0.05 was considered to be significant.

## 3. Results

Patient characteristics are presented in Table 1.

The median patient age at time of operation was 65 years, and the median BMI was 23.6. One hundred thirty-three (49%) patients had a right-sided tumor, whereas 139 (51%) patients had a left-sided tumor. The clinical T stage was cT1a in 193 (71%) patients and cT1b in 79 (29%) patients. The mean RENAL nephrometry score was 7. Median tumor diameter was 30 mm. RN was performed in 170 (63%) patients, whereas PN was performed in 102 (37%) patients. Overall, the median operative time was 197 min. During the median follow-up period of 35 months, five (2%) patients died. Sixteen (6%) showed recurrence (four local and 12 distant metastasis).

Pathological T3a upstaging (pT3a group) occurred in 33 (12%) patients. The remaining 239 (88%) were diagnosed as pathological T1 (pT1 group). The preoperative characteristics of the pT3a group and the pT1 group are presented in Table 2.

There was no significant difference between the groups with respect to age at operation, gender, tumor laterality, BMI, and RENAL nephrometry score.

The clinical T1b stage was significantly more prevalent in the pT3a group than the pT1 group (64% vs. 24%, *p* < 0.0001). The tumor diameter was significantly larger in the pT3a group than in the pT1 group (50 mm vs. 30 mm, *p* < 0.0001). The preoperative AST/ALT ratio was significantly higher in the pT3a group than in the pT1 group (1.13 vs. 1.09, *p* = 0.037). The CRP levels were also significantly higher in the pT3a group than in the pT1 group (0.1 vs. 0.09, *p* = 0.014). There was no significant difference in terms of preoperative AST and ALT levels. The results of multivariate regression analyses of preoperative predictive factors for pathological T3a upstaging of clinical T1 RCC are shown in Table 3.

Upon multivariate analyses, tumor diameter (<30 mm vs. ≥30 mm) was the only significant preoperative predictor of pT3a upstaging [hazard ratio (HR), 3.61; 95% confidence interval (CI), 1.32–9.84; *p* = 0.01]. The AST/ALT ratio (<1.1 vs. ≥1.1) tended to be a preoperative predictor of pT3a upstaging, although it was not significant (HR 2.14; 95% CI: 0.97–4.73; *p* = 0.06).

Pathological T3a upstaging occurred in 25% of patients with both a preoperative tumor diameter ≥30 mm and a preoperative AST/ALT ratio ≥1.1. There was a significant correlation between pathological T3a upstaging and the number of preoperative risk factors (chi-square, *p* = 0.0002) (Table 4).

The surgical and histopathological data of the pT3a and pT1 groups are presented in Table 5.

The pT3a group had a higher proportion of RN compared to the pT1 group (100% vs. 57%, *p* < 0.0001). High grade tumors (Fuhrman grade 3–4) were significantly more common in the pT3a group compared to the pT1 group (24% vs. 4%, *p* < 0.0001). Microscopic venous invasion (v+) and microscopic lymphatic invasion (ly+) were also significantly more prevalent in the pT3a group compared to the pT1 group (64% vs. 5%, *p* < 0.001; 15% vs. 0%, *p* < 0.0001, respectively). There was no significant difference in tumor histology between the groups (*p* = 0.22).

**Figure 1:**
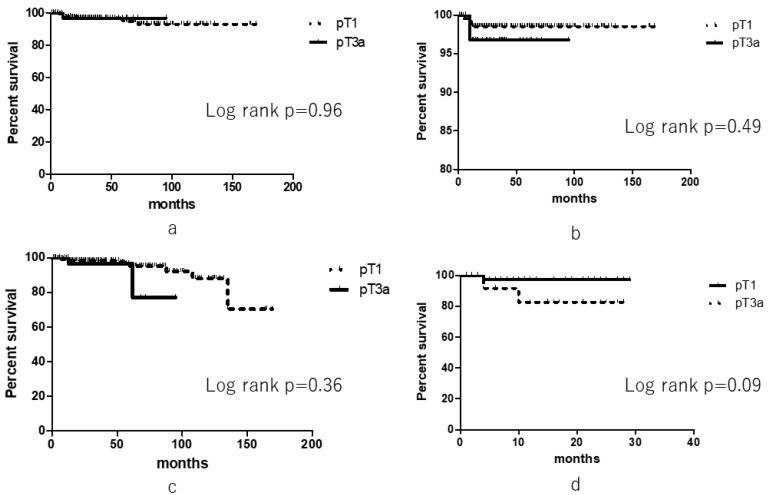
Oncological outcomes of pathological T3 upstaging and pathological T1 patients. (**a**) Overall survival, (**b**) Cancer-specific survival, (**c**) Recurrence-free survival, (**d**) Two-year recurrence-free survival).

With respect to outcomes, there were no significant differences between the groups in terms of OS (log rank, *p* = 0.96), CSS (log rank, *p* = 0.49), and RFS (log rank, *p* = 0.36). There was a difference in terms of two-year RFS rate between the groups, although it was not significant (82.5% vs. 97.3%, *p* = 0.09) (Figure 1).

## 4. Discussion

There has been an increase in the detection of incidental small renal masses due to advances in diagnostic imaging technologies. PN for organ-confined small renal masses has been the standard treatment option because it provides an extension of OS, preserves renal function, and reduces cardiovascular risk compared to RN [5,6]; PN also provides similar oncological outcomes as RN [5,6,7]. However, in locally advanced RCC, the resection of Gerota’s fascia and the dissection of the perirenal fat during PN may increase the risk of recurrence [8].

The TNM classification of a tumor is essential in order to determine its appropriate treatment, surgical technique, follow-up protocol, and oncological prognosis [9,10]. Clinical T1 and T2 tumors are classified according to tumor size. Tumors with diameter of ≤4 cm are defined as clinical T1a, ones diameters from 4< to ≤7 cm are defined as clinical T1b, and diameters more than 7 cm with organ confined tumors are defined as clinical T2 tumors. However, clinical T3a tumors are defined by the presence of peripheral fat invasion, renal sinus fat infiltration, or renal vein extension regardless of tumor size. Though contrast-enhanced CT is commonly used diagnostically for segmental or main renal vein invasion, its reported sensitivity is only 59–69% [11]. Sokhi et al. reported the sensitivity of CT for identifying the tumor invasion of the renal sinus fat (88%), perirenal fat (83%), and renal vein (69%) [12]. Microscopic perirenal invasion, renal sinus fat infiltration, or renal vein extension can be missed with contrast-enhanced CT imaging, and pathological T3a upstaging occasionally occurs in patients with cT1 tumors.

Previous studies have reported a poor prognosis in RCC patients with cT1 tumors upstaged to pT3a when compared to patients with pT1 tumors. Gorin et al. reported the incidence and outcomes of pathological T3a upstaging in patients with clinical T1 RCC who underwent robotic PN [1]. Pathological T3a tumors were observed in 4.8%, and the 24-month RFS rates for pT1-2 and pT3a tumors were 99.2% and 91.8%, respectively. Jeong et al. reported that pT3a upstaging occurred in 9.2% of cT1 RCCs, and upstaging was associated with a significantly poorer prognosis compared to pT1 disease; the two-year RFS rates in the pT3a upstaged and pT1 groups were 87.3% and 98.7%, respectively (*p* < 0.001) [2]. Ghanie et al. [3] also analyzed the pathological upstaging of clinical T1 RCC using the National Cancer Data Base Participant User File. They reported that pT3a upstaging was observed in 5.4% patients with cT1 RCC and that upstaging led to a 40% increase in risk of death compared to patients with pT1 tumors. Russel et al. also reported significantly reduced CSS and RFS rates in patients with pT3a upstaging following PN compared to pT1 tumors [4]. In our study, however, there was no significant difference between the pT3a and pT1 groups in terms of OS, CSS, and RFS.

Previous reports have analyzed predictive factors for pT3a upstaging. Gorin et al. reported that preoperative predictive factors for pT3a upstaging were tumor diameter and location [1]. Similarly, Jeong et al. noted older age, cT1b stage, clinical symptoms, and a high Fuhrman grade as such [2]. Ghanie et al. reported that the predictive factors for pT3a upstaging were patients who were older, male, had comorbidities, had cT1b tumors, underwent RN, and had a high Fuhrman grade [3]. Ramaswamy et al. [13] demonstrated that T3a upstaging was associated with a clear cell histology, tumor size >4 cm, and a positive surgical margin on pathological examination. Tay et al. [14] found that a high RENAL nephrometry score was a predictive factor for pT3a upstaging, but age and Fuhrman grade were not.

As noted above, the previous reports tended to describe the relationship between histopathological findings and pT3a upstaging. We wanted to investigate preoperative predictive factors for pT3a upstaging, because they may affect the surgical approach or technique in patients with clinical T1 RCC.

In our study, the preoperative predictive factors for pathological T3a upstaging were tumor diameter, CRP level, and AST/ALT ratio in univariate analysis. A multivariate analysis showed that tumor diameter was the only significant preoperative predictor of pathological T3a upstaging; the AST/ALT ratio tended to be a predictor but it was not significant. When the preoperative tumor size and AST/ALT ratio were further considered in detail, pathological T3a upstaging occurred in 25% of patients with both tumor diameter ≥3.0 mm and an AST/ALT ratio ≥1.1.

Aminotransaminases, including AST and ALT, are well-known liver enzymes produced by both malignant and non-malignant cells, and they are blood-based circulating biomarkers. ALT is only existent in the hepatocellular cytoplasm and mitochondria. AST, on the other hand, is widely spread in several organs, including the heart, kidney, brain, skeleton, muscle, and liver [15]. The functions of AST and ALT represent crucial metabolic interactions between protein and carbohydrate metabolism. AST and ALT are also important in all cells that have a high metabolic activity; AST is particularly vital for aerobic glycolysis [15]. Malignant cells show a higher rate of glycolysis than non-malignant cells [16]. Pathological processes that can lead to a higher proliferative state, tissue damage, and high tumor cell turnover tend to increase AST but not ALT, making the AST/ALT ratio an attractive potential biomarker.

Previous reports have demonstrated that these enzymes could be a significant prognostic biomarker in several malignancies such as those of the lung, colon, pancreatic, and breast cancer [17,18,19,20].

The AST/ALT ratio has been widely used in the evaluation of liver disease. Moreover, it could also be a crucial prognostic factor in various malignancies [21,22,23]. Bezan et al. [24] reported that the preoperative AST/ALT ratio is an independent prognostic factor in patients with non-metastatic RCC; a high AST/ALT ratio was significantly associated with poor outcomes regarding progression-free survival (PFS) and OS. Lee et al. [25] also reported that an elevated AST/ALT ratio was significantly associated with worse postoperative survival in patients surgically treated for non-metastatic RCC; a high AST/ALT ratio was significantly associated with poor PFS, CSS, and OS. Canat et al. reported [11] a significant correlation between the AST/ALT ratio and tumor histopathological variables and demonstrated that RCC patients with a high preoperative AST/ALT ratio were more likely to have renal vein invasion observed on histopathological examination. Venous invasion is said to be a poor prognostic factor and associated with poor survival rates. Though CT is commonly used as diagnostic imaging for segmental or main renal vein invasion, the reported sensitivity of this modality is reportedly only 59–69%; the AST/ALT ratio may be considered a sensitive biomarker for the prediction of renal vein invasion [11].

Jeong et al. [2] found that surgical technique (PN or RN) did not affect the recurrence rate for cT1 RCC upstaged to pT3a. Capitanio et al. [26] also reported no difference in terms of metastatic PFS and CSS between PN and RN cohorts in patients upstaged to pathological T3a RCC disease. They concluded that cancer control is similar between patients treated with an extirpation of the entire kidney and those with partial resection, even if the final histopathological examination demonstrated a tumor that was unexpectedly upstaged from clinical T1 to pathological T3a.

On the other hand, Shah et al. [27] reported that those who undergo PN appear to have inferior RFS relative to those who undergo RN. They reviewed the records of 1250 patients with clinical T1 RCC who underwent partial or radical nephrectomies and showed that pathological T3a upstaging was noted in 140 patients (11%); among the pathological T3a upstaged patients, PN was associated with shorter RFS compared to RN.

Controversy remains regarding whether surgical approach (PN or RN) affects the recurrence rate in RCC. We believe that attention should be paid to the resection of perirenal fat and renal parenchyma during surgery in patients with clinical T1 RCC who have a large tumor and a high preoperative AST/ALT ratio, because these tumors may have a higher possibility to be upstaged to pT3a.

There are some limitations to this study. It is retrospective in nature with a limited follow-up period and examined a relatively small number of patients. However, to the best of our knowledge, this is the first study showing the correlation between the preoperative AST/ALT ratio and pathological T3a upstaging in patients with clinical T1 RCC. Further prospective studies are needed to validate our findings.

## 5. Conclusions

In conclusion, this study showed that larger tumors and probably preoperative high AST/ALT ratio were significantly associated with pathological T3a upstaging in patients with clinical T1 RCC, suggesting that they are potential preoperative predictive factors.

## Figures and Tables

**Table 1:** Characteristics of the patients (*n* = 272).

Age (Years, median, range)		65 (33–85)
gender (n, %)	male	196 (72)
	female	76 (28)
laterality (n, %)	right	133 (49)
	left	139 (51)
BMI (median, range)		23.6 (15.5–38.9)
clinical stage (n, %)	cT1a	193 (71)
	cT1b	79 (29)
RENAL score (median, range)		7 (4–10)
Tumor diameter (mm, median, range)		30 (8–70)
operation (n, %)	radical nephrectomy	170 (63)
	partial nephrectomy	102 (37)
procedure (n, %)	laparoscopic surgery	144 (53)
	open surgery	128 (47)
operative time (min, median, range)		197 (90–547)
pathological stage (n, %)	pT3a	33 (12)
	pT1	239 (88)
follow-up (months, median, range)		35 (1–169)
outcome (n, %)	survive	248 (91)
	recurrence	16 (6)
	death	13 (5)
	cancer-death	5 (2)

**Table 2:** The differences of the preoperative parameters between pT3a and pT1 groups.

	pT3a (*n* = 33)	pT1 (*n* = 239)	*p*-Value
age (years, median, range)	64 (42–85)	65 (33–84)	0.06 *
gender (n, %)			
male	25 (76)	171 (72)	0.68 **
female	8 (24)	68 (28)	
laterality (n, %)			
right	16 (48)	117 (49)	1.00 **
left	17 (52)	122 (51)	
BMI (median, range)	16 (15.5–38.9)	19 (17.3–37.0)	0.076 *
Clinical stage (n, %)			
cT1a	12 (36)	181 (76)	<0.0001 **
cT1b	21 (64)	58 (24)	
RENAL score (median, range)	7 (5–9)	6 (4–10)	0.10 *
Tumor diameter (mm, median, range)	50 (21–70)	30 (8–70)	<0.0001 *
AST (IU/L, median, range)	16.0 (12–86)	19.0 (5–82)	0.076 *
ALT (IU/L, median, range)	14.5 (6–96)	16.0 (4–286)	0.24 *
AST/ALT ratio (median, range)	1.13 (0.64–2.93)	1.09 (0.28–2.83)	0.037 *
CRP (mg/dL, median, range)	0.1 (0.01–11.6)	0.09 (0.01–6.99)	0.014 *

* The Mann–Whitney test, ** Chi-square test.

**Table 3:** Multivariate analysis for pathological T3a upstaging.

	HR	95% CI	*p*-Value
Tumor diameter (<30 vs. ≥30 mm)	3.61	1.32–9.84	*p* = 0.01
AST/ALT ratio (<1.1 vs. ≥1.1)	2.14	0.97–4.73	*p* = 0.06
CRP (<0.1 vs. ≥0.1)	1.22	0.55–2.72	*p* = 0.61

**Table 4:** The number of preoperative risk factors and the incidence of pathological T3a upstaging.

	cT1 (*n* = 272)	pT3a (*n* = 33)	pT1 (*n* = 239)	*p*-Value
Preoperative risk factors (tumor diameter, AST/ALT ratio)				*p* = 0.0002
2 factors	73	18 (25%)	55 (75)	
1 factor	155	14 (9)	141 (91)	
0 factor	44	1 (2)	43 (98)	

**Table 5:** The differences of the tumor characteristics and postoperative parameters between the pT3a and pT1 groups.

	pT3a (*n* = 33)	pT1 (*n* = 239)	*p*-Value
Type of operation (n, %)			
radical nephrectomy	33 (100)	137 (57)	<0.0001 ***
partial nephrectomy	0 (0)	102 (43)	
Procedure (n, %)			
laparoscopic surgery	27 (83)	117 (49)	0.0005 ***
open surgery	6 (17)	122 (51)	
Tumor histology (n, %)			
clear cell RCC	30 (91)	194 (81)	0.22 ***
non-clear cell RCC	3 (9)	45 (19)	
Fuhrman grade (n, %)			
1	3 (9)	58 (24)	<0.0001 **
2	20 (61)	151 (63)	
3	8 (24)	8 (3)	
4	0 (0)	2 (1)	
unclear	2 (6)	20 (8)	
INF (n) a/b/c/ unclear	13/20/0/0	178/48/0/13	<0.0001 **
v + (n, %)	21 (64)	11 (5)	<0.0001 ***
ly + (n, %)	5 (15)	0 (0)	<0.0001 ***
Follow-up (months, median, range)	27 (6–95)	35 (1–169)	0.55 *
Outcome (n, %)			
survive	29 (88)	219 (92)	0.78 **
Recurrence	3 (9)	13 (5)	
Death	2 (6)	11 (5)	
Cancer-death	1 (3)	4 (2)	

* The Mann–Whitney test, ** Chi-square test, *** Fisher’s exact test.
